# Glia-derived VCAM1 promotes glioma progression

**DOI:** 10.3389/fonc.2026.1802953

**Published:** 2026-04-24

**Authors:** Jiajing Dai, Hailong Zheng, Jiaxu Luo, Minlin Dai, Xiao-Ling Hu, Wenzhi Sun

**Affiliations:** 1 Department of Neurobiology, Basic Medical Sciences, Capital Medical University School, Beijing, China; 2 Chinese Institute for Brain Research, Beijing, China

**Keywords:** astrocyte, glioma, glioma stem cell-like cells, therapeutic target, VCAM1

## Abstract

**Background:**

The dynamic interactions between glioma cells and the tumor microenvironment (TME) drive tumor progression and therapeutic resistance. VCAM1 is known to facilitate tumor metastasis in various cancers; however, its specific function in the glioma microenvironment remains poorly defined.

**Methods:**

To elucidate VCAM1’s role in glioma, we evaluated its expression in human glioma datasets and correlated it with patient survival outcomes. Using syngeneic and primary mouse glioma models, we characterized VCAM1 expression in tumor and stromal cell populations. Furthermore, we selectively ablated VCAM1 in GLAST-positive astrocytes in a glioma-bearing mouse model to evaluate its functional impact on tumor growth and overall survival.

**Results:**

We identified that VCAM1 is highly enriched in proliferative glioma stem cell-like cells (GSLCs); for example, 86.5% of SOX2^+^, 86.3% of CD133^+^ and 93.4% of Ki67^+^ cells co-expressed VCAM1 in the 73C glioma model. In the normal brain, VCAM1 is largely restricted to the vasculature, but it is notably expressed in TME-associated astrocytes. Crucially, the selective deletion of astrocytic VCAM1 significantly extended the median survival of glioma-bearing mice, such as from 52 to 66.5 days in a hippocampal GL261 model. However, this survival benefit depends on tumor location and genetic background, showing limited efficacy against aggressive hippocampal 73C tumors. Clinical data aligned with these findings, showing that lower VCAM1 expression correlates with prolonged survival in low-grade glioma patients, but not in glioblastoma.

**Conclusion:**

Astrocyte-derived VCAM1 is a critical driver of glioma progression, mediating essential interactions between tumor cells and the TME. Targeting VCAM1 signaling presents a promising, microenvironment-focused therapeutic strategy, though its clinical application must account for regional and genetic tumor heterogeneity.

## Introduction

GBM is a devastating primary brain tumor characterized by rapid progression, profound intra-tumoral heterogeneity and a grim prognosis, with a median survival of only 12–18 months (1, 2). Despite the standard-of-care, comprising surgery, radiotherapy, and temozolomide (TMZ) chemotherapy (3, 4), tumor recurrence is nearly inevitable. This treatment failure is largely driven by remodeling of the extracellular matrix (ECM), glioma stem cell-like cells (GSLCs) and immune evasion (5–9). Within the TME, astrocytes engage in dynamic interactions with glioma cells to promote tumor growth, invasion and immune evasion (10).

Astrocytes, a major cellular component of the central nervous system, are heavily recruited into the glioma TME. Reactive astrocytes provide critical metabolic and structural support to expanding tumors (11, 12). However, the specific adhesion mechanisms that physically anchor glioma cells to the astrocytic niche and mediate these pro-tumorigenic interactions remain incompletely characterized.

To identify novel regulators of glioma malignancy, we analyzed transcriptomic and clinical survival data from the glioma cohort of The Cancer Genome Atlas (TCGA), revealing that elevated expression of Vascular Cell Adhesion Molecule-1 (VCAM1) is associated with shorter overall survival in low-grade glioma (LGG). VCAM1 is a key adhesion molecule of the immunoglobulin superfamily, classically known for mediating leukocyte-endothelial adhesion during inflammation (13, 14). Recent studies have also highlighted VCAM1’s role in solid tumors, for instance, VCAM1 facilitates breast cancer bone metastasis by attracting osteoclast progenitors and promoting osteoclast activity (15).

Despite these findings, a critical research gap remains regarding VCAM1’s role in glioblastoma. Existing research has predominantly focused on VCAM1 expression in vascular endothelial cells. Whether VCAM1 is expressed by other critical TME populations, such as astrocytes or GSLCs, and how it functionally contributes to glioma progression within the unique brain microenvironment, is largely unknown. Elucidating VCAM1’s specific cellular sources and functions could offer valuable insights into glioma biology and reveal microenvironment-specific therapeutic targets.

In this study, we investigate the precise cellular distribution and functional role of VCAM1 in glioblastoma progression. We hypothesize that astrocyte-derived VCAM1 mediates critical adhesion and signaling within the TME, representing a potential therapeutic target.

## Methods

### Glioma cell lines

Three glioma cell lines were utilized in this study, GFP^+^73C, GL261-luc, and GFP^+^GL261-luc. GFP^+^73C glioma cells containing mutations including p53^-/-^,Pten^-/-^,and Braf^V600E^, originating from primary mouse astrocytes, were sourced from the laboratory of Woo-ping Ge. GL261-luc glioma cells have luciferase reporter *luc*, obtained from the laboratory of Jian Chen. GL261-luc cells were infected with AAV of pHAGE-3F-ccDB-GFP to obtain GFP^+^GL261-luc cells. All cell lines were grown in DMEM with 10% fetal bovine serum (FBS) supplementation, and three cell lines were cultured in humidified incubator at 37 °C with 5% CO_2_ and passaged to sustain 80%-90% confluency.

### Mice and genotyping of mouse lines

#### Mice

All mice were housed and bred on a 12/12 light/dark cycle in the specific pathogen-free vivarium of Chinese Institute for Brain Research (CIBR), Beijing, with ad libitum access to food and water. Eight- to twelve-week-old mice of both sexes were used in the study. All experimental procedures were approved by the Institutional Animal Care and Use Committee at Capital Medical University and the Chinese Institute for Brain Research.

The mice used in this study included adult C57BL/6J, Ai14, VCAM1-CreER, VCAM1-CreER::Ai14, GLAST-CreER, GLAST-CreER::Ai14, GLAST-CreER::VCAM1flox/flox, and GLAST-CreER::VCAM1*^fl/fl^*::Ai14. C57BL/6J (stock#000664), Ai14 line (stock #007914), GLAST-CreER line(stock#012586), VCAM1*^fl/fl^* (stock#007665) were purchased from Jackson Laboratory. VCAM1-CreER were obtained from cyagen (Suzhou, China), the TAG stop codon (in exon9) were replaced with the “2A-CreER” cassette. To generate VCAM1-CreER::Ai14 mice and GLAST-CreER::Ai14 mice, Ai14 mice were crossed with VCAM1-CreER mice and GLAST-CreER mice, respectively. To generate GLAST-CreER::VCAM1*^fl/fl^* mice, VCAM1*^fl/fl^* mice were crossed with GLAST-CreER to generate GLAST-CreER::VCAM1*^fl/+^* mice that were further crossed with littermate heterozygous mice to generate GLAST-CreER:: VCAM1*^fl/fl^*. To generate GLAST-CreER:: VCAM1*^fl/fl^*::Ai14, Ai14 mice were crossed with CreER:: VCAM1*^fl/fl^* to generate GLAST-CreER::VCAM1*^fl/+^*::Ai14, the intermediate lines was further crossed with VCAM1*^fl/fl^* or CreER::VCAM1*^fl/+^*::Ai14 to generate GLAST-CreER::VCAM1*^fl/fl^*::Ai14. Experiments involving astrocytes specific VCAM1-/- were performed using age-matched littermates of either sex.

#### Genotyping of mouse lines

For genotyping methods, primer sequences and PCR conditions see below.

Ai14 (Ai14-WT-9020:5-AAGGGAGCTGCAGTGGAGTA-3; Ai14-WT-9021:5-CCGAAAATCTGTGGGAAGTC-3; Ai14-Mut-9103:5-GGCATTAAAGCAGCGTATCC-3; Ai14-Mut-9105:5-CTGTTCCTGTACGGCATGG-3; PCR products: mut band:196bp, WT band:297bp); VCAM1-CreER (VCAM1CreER-F:5-AGTGAAATCTCCAAAGACCATCAGT-3; VCAM1CreER-R:5-TAGCCATGAACAGAAATGCCTAAC-3; PCR products: mut band:305bp, WT band:222bp); GLAST-CreER (GLASTCreER-Ctrl-8744:5-CAAATGTTGCTTGTCTGGTG-3; GLASTCreER-Ctrl-8745:5-GTCAGTCGAGTGCACAGTTT-3; GLASTCreER-Trans-10110:5-ACAATCTGGCCTGCTACCAAAGC-3; GLASTCreER-Trans-10112:5-CCAGTGAAACAGCATTGCTGTC-3; PCR products: mut band:600bp, WT band:200bp); VCAM1flox/flox(VCAM1flox-F:5-GGGACGGATTTTCTTTCCAC-3; VCAM1flox-R:5-GACTTTGAAGCCCATTGCAC-3; PCR products: mut band:226bp, WT band:162bp).

Mouse toe tissues were lysed using one step mouse genotyping kit (Vazyme#PD101-01), with each sample immersed in 100μL lysis buffer containing 2% Proteinase K, followed by an incubation at 55 °C for 30 minutes and then at 95 °C for 5 minutes. And 1μL of the lysate was used as the DNA template for PCR amplification. PCR conditions for all PCRs: one cycle at 94°C for 5min; 10 cycles at 94°C for 20s, 65°C for 30s(decrease the temperature by 0.5 °C per cycle), 68°C for 30s; 28 cycles at 94°C for 20s, 60°C for 20s, 72°C for 30s; and then followed by 72°C for 5min. Genotyping results of these mouse lines for these mouse lines showed in **Supplementary Figures**.

### Cell sorting by flow cytometry

GL261-luc tumor cells were collected 72 hours after infection with AAV (pFUGW-EGFP), and the cell suspension was prepared following standard cell culture procedures. eGFP-positive GL261-luc cells were sorted using a flow cytometer (FACSAriaII, 100 μm nozzle) with single-positive GL261-luc cells used for voltage adjustment and gating to isolate eGFP-positive GL261-luc cells. The sorted eGFP-positive GL261-luc cells were then seeded into culture dishes for further expansion.

### Stereotaxic injection of glioma cells

To inoculate each mouse model with 4000 tumor cells, cell counting was performed using AO/PI staining with a cell counter. It was ensured that the duration of placing each batch of implanted tumor cells on ice did not exceed two hours to minimize variability among the model mice. A stereotactic apparatus was used for injecting glioma cells. Mice were anesthetized using a gas anesthesia mixture of 2% isoflurane and 98% O_2_ mixture for maintenance on the mouse surgical table. A heating pad was placed on the surgical table to maintain the temperature at 37°C. Apply erythromycin ointment to the mouse’s eyes to protect them from the cold light source and exposing the skin on the head. The height difference between the anterior and posterior fontanelles of the mouse head was used to adjust the position of the mouse head, ensuring accurate localization for brain region injection. The height difference between the anterior and posterior fontanelles should not exceed 0.1mm to ensure accurate positioning. Using a micro syringe pump, 4000–5000 tumor cells were injected into the targeted brain area of each mouse at a rate of 5nL/s. The injection of V1 cortex was performed at the following coordinates: Anterior-Posterior (AP) -2.5mm, Medial-Lateral (ML) 2.5mm, Dorsal-Ventral (DV) 1.0mm; for the hippocampus: AP -2.0mm, ML1.5mm, DV 2mm. After injection, the needle was held in place for over 15 minutes to ensure no leakage of tumor cells. Following the procedure, bone wax was used to seal the skull holes, and the incision on the mouse’s scalp was sutured. The mice were then allowed to recover from anesthesia.

### Tamoxifen preparation and treatment

Tamoxifen was dissolved with corn oil (10mg/ml). Mice were administered tamoxifen with 150μl/20g body weight per day. GLAST-CreER::VCAM1*^fl/fl^* mice were administered tamoxifen for seven consecutive days and then wait for another seven days before stereotactic injection of GL261-luc tumor cells. VCAM1-CreER::Ai14 mice were injected GL261-luc tumor cells and administered tamoxifen on the same day, followed by intraperitoneal injections of tamoxifen for six days.

### Immunobiological staining

At the pre-scheduled time after implantation of tumor cells, the mice were anesthetized via intraperitoneal injection with 2.5% avertin (14μl/g body weight) and subjected to perfusion with phosphate-buffered saline (PBS) to clear the blood from the circulatory system, followed by a secondary perfusion with a solution of 4% paraformaldehyde (PFA) dissolved in PBS. The mice brains were extracted, post-fixed overnight in 4% PFA, and then dehydrated using a 30% sucrose solution. Following dehydration, the brain tissue samples were embedded in optimal cutting temperature (OCT) compound and sectioned into 40μm slices using a cryostat (model CM3050 S, Leica). These slices were then mounted onto adhesive glass slides. Immunostaining procedures were carried out on free-floating sections with a 1-hour incubation in a blocking solution at room temperature. This solution was a mixture of 3% bovine serum albumin (BSA), 2% goat serum and 0.3% Triton X-100 in PBS. Subsequently, the incubation with primary antibodies was carried out at 4 °C overnight. Primary antibodies diluted in the blocking solution included rabbit anti-KI67(Abcam;1:500;ab15580), rabbit anti-CD133 antibody (Abcam; 1:500; ab19898), rat anti-Nestin antibody (DSHB; 1:10; rat-401), rat anti-Vcam1 antibody (BD; 1:50; 550547), rat anti-PDGFRβantibody (Thermo; 1:300; 14-1402-81), rabbit anti-Sox2 antibody(Abcam; 1:500; ab97959), rabbit anti GFAP antibody (Sigma-Aldrich; 1:300; G9269), rabbit anti-Laminin antibody (Sigma-Aldrich; 1:300; L9393), rabbit anti-Glut1 antibody (Millipore; 1:300; 07-1401), rabbit anti-S100βantibody (Abcam; 1:400; ab52642). Following triple washing with 1×PBS, the sections were treated with secondary antibodies for 1 hour at room temperature. Secondary antibodies (1:1000 in 1×PBS) included goat anti-rabbit IgG conjugated to Alexa Fluor488; goat anti rat IgG conjugated to Alexa Fluor 647; donkey anti rabbit IgG conjugated to Alexa Fluor 647. Another triple washing with 1×PBS after secondary antibodies before stained in DAPI or Hoechst solution with 1-5 µg/ml. Sections were cover-slipped using Vectashied mounting medium (Vector Laboratories), and finally sealed with nail polish to prevent the medium from drying out or being dislodged. Images were obtained using a Leica SP8 confocal microscope, where specific excitation wavelengths were applied for confocal imaging of different fluorochromes: 405nm for DAPI, 488nm for GFP, 552nm for tdTomato, and 638nm for Alexa Fluor 647.

### Acute cell staining

To facilitate the identification of cell subpopulations expressing VCAM1 within tumor tissues, 5000 eGFP^+^ GL261-luc glioma cells were transplanted into the hippocampus, and after 20 days, as well as 5000 eGFP^+^ 73C tumor cells after 12 days post-transplantation, the tumor tissues were isolated and dissociated into single cells. The tumor single-cell suspension was then plated onto cover slips that had been pre-treated with poly-L-lysine (PLL) to avoid *in vitro* differentiation. To prevent such differentiation, the tumor single cells were immediately fixed with 4% paraformaldehyde (PFA) for 20 minutes after adhering to the cover slips for 3 hours. After the completion of staining, the cover slips were removed and mounted upside down onto glass slides.

### Live imaging analysis

We conducted live imaging analysis using PerkinElmer IVIS Lumina LT III. For mouse anesthesia, a 5% isoflurane and 95% O_2_ mixture was used for induction in a plexiglass chamber, followed by a 2% isoflurane and 98% O_2_ mixture for maintenance inside the casing of the device. We used D-luciferin firefly, potassium salt (Yushen Biology; 40902ES03) as the substrate for luciferase, at an injection of concentration of 150mg/kg (200ul/20g) for intraperitoneal injection into mice. Imaging analysis was performed 10–15 minutes after injection into the mice.

### Isolation of primary cells from mouse brain

The mouse was perfused with hibernation medium (HIB), and the brain tissue was isolated and immersed in HIB. On ice, the target brain regions were dissected under a stereomicroscope. The brain tissue was dissociated into approximately 1mm fragments using forceps, and the tissue pellet was collected. 3-5ml of digestion solution is required for each mouse brain region. The digestion solution is prepared by adding papain at a final concentration of 2% in DMEM, and the tissue is digested at 37 °C for 30 min. Gently triturate the sample until a single-cell suspension is obtained, then add 10% Opti-prep and centrifuge at 1300×g for 5min. Resuspend the cell pellet in HIB, and the prepared single-cell suspension is then used for flow cytometry sorting.

### RNA-seq of tdTomato+ cells

tdTomato^+^ cells of GLAST-CreER::VCAM1*^fl/fl^*::Ai14 mice and tumor-bearing GLAST-CreER::VCAM1*^fl/fl^*::Ai14 mice were sorted by flow cytometry. During the process of isolating the tdTomato^+^ cells, the proportion of live cells decreased over time due to flow cytometric sorting. Instead of RNA extraction, we used the method of collecting 1000 cells directly into lysis buffer, followed by first-strand cDNA synthesis and amplification to obtain sufficient material for sequencing analysis. The experimental protocol for constructing single-cell transcriptome sequencing libraries followed the protocol of the Discover-sc™ WAT Kit V2 (Vazyme). RNA-seq libraries were constructed and sequenced on a HiSeq X Ten platform (Illumina, USA) generating 150-bp paired-end reads by AZENTA (Suzhou, China).

### RNA-seq data analysis

We conducted quality control of the original read files using FastQC (version 0.12.1) and filtered low-quality reads and adapter sequences induced by the tagmentation library using Trimmomatic (version 0.39) (16). For reads alignment, we used the reference genome GRCm38.p6 from the Genome Reference Consortium and genome annotation (version M23) from GENCODE, mapping RNA-seq reads with STAR (version 2.7.11a) (17). To generate a sample-gene count matrix, we assigned mapped reads using featureCounts (version 2.0.6) (18). Subsequent analyses were performed primarily in R. To correct for batch effects, we used the combat function from the sva package (version 3.46) (19), incorporating company type as a covariate. Principal component analysis (PCA) was conducted using factoextra (version 1.0.7) and FactoMineR (version 2.8). For data normalization, we calculated the Fragments Per Kilobase of transcript per Million mapped reads (FPKM). FPKM was strictly utilized because it intrinsically accounts for both sequencing depth and gene length, ensuring accurate normalization and reliable comparison of gene expression levels across our paired-end sequencing samples. Subsequently, differential gene expression analysis was performed using the limma package (version 3.54.2) (20). Genes were defined as significantly differentially expressed based on strict filtering criteria: an absolute log2 fold change (|log2FC|)≥1 and a *p* < 0.05. Functional enrichment analyses, including KEGG and GO enrichment, were conducted with clusterProfiler (version 4.6.2) (21). Finally, visualization of transcriptome analysis results was performed using ggplot2 (version 3.5.0).

### Data analysis and statistics

Statistical analyses were performed using GraphPad Prism software and R. Quantitative data are presented as the mean ± standard error of the mean (SEM). For simple comparisons between two independent groups (such as cell proportions and relative fluorescence intensity), an unpaired, two-tailed Student’s t-test was utilized. For the longitudinal quantification of tumor burden via *in vivo* bioluminescence imaging, a two-way repeated-measures analysis of variance (ANOVA) was performed to evaluate the interaction between time and genotype. Animal survival rates were plotted using Kaplan-Meier curves, and statistical differences between groups were determined using the Log-rank Mantel-Cox test. For RNA-seq data analysis, differential gene expression was evaluated using the limma package, with statistical significance strictly defined as an absolute log2 fold change (|log2FC|) ≥ 1 and a p < 0.05. Across all general analyses, a p<0.05 was considered statistically significant. Asterisks denote statistical significance: *p < 0.05, **p < 0.01, ***p < 0.001, ****p < 0.0001.

### Study design and reproducibility

All *in vitro* and *in vivo* experiments were independently repeated to ensure reproducibility. For animal studies, sample sizes were determined based on standard protocols for glioma models to ensure adequate statistical power. Specifically, n = 6 to 7 mice were used per experimental or control group for all survival and tumor growth tracking assays. Age- and sex-matched littermates were randomly assigned to their respective groups prior to stereotaxic tumor cell implantation. For histological and immunofluorescence quantifications, multiple microscopic fields (typically 6 fields derived from 3 independent mice per group) were randomly selected for blinded quantification. For the RNA-sequencing analysis, n = 6 biological replicates per group (derived from 3 mice per group, 2 independent samples per mouse). Data acquisition (such as IVIS imaging) and histological quantitative analyses were performed objectively to minimize potential observer bias.

## Results

### VCAM1 expression was enriched in a proliferative stem-like tumor cell population in syngeneic mouse models of glioblastoma

To characterize VCAM1 expression and intratumoral heterogeneity, we employed two syngeneic murine glioblastoma models: eGFP^+^ GL261-luc (*Trp53-/-* and *K-ras*-/-) *(*22, 23) and eGFP^+^ 73C (*Trp53*-/-, *Pten*-/-, *Braf* V600E) (24). In both cell lines, VCAM1 was preferentially expressed in tumor cells exhibiting a proliferative, stem cell-like phenotype. Specifically, the majority of VCAM1^+^cells co-expressed the GSCs markers SOX2, CD133 and Nestin, as well as the proliferation marker Ki67 (**Supplementary Figures 1A, S1B**). This finding suggests that VCAM1 is enriched within the GSLC compartment, a cell population widely implicated in glioma pathogenesis and treatment failure (25, 26).

**Figure 1:**
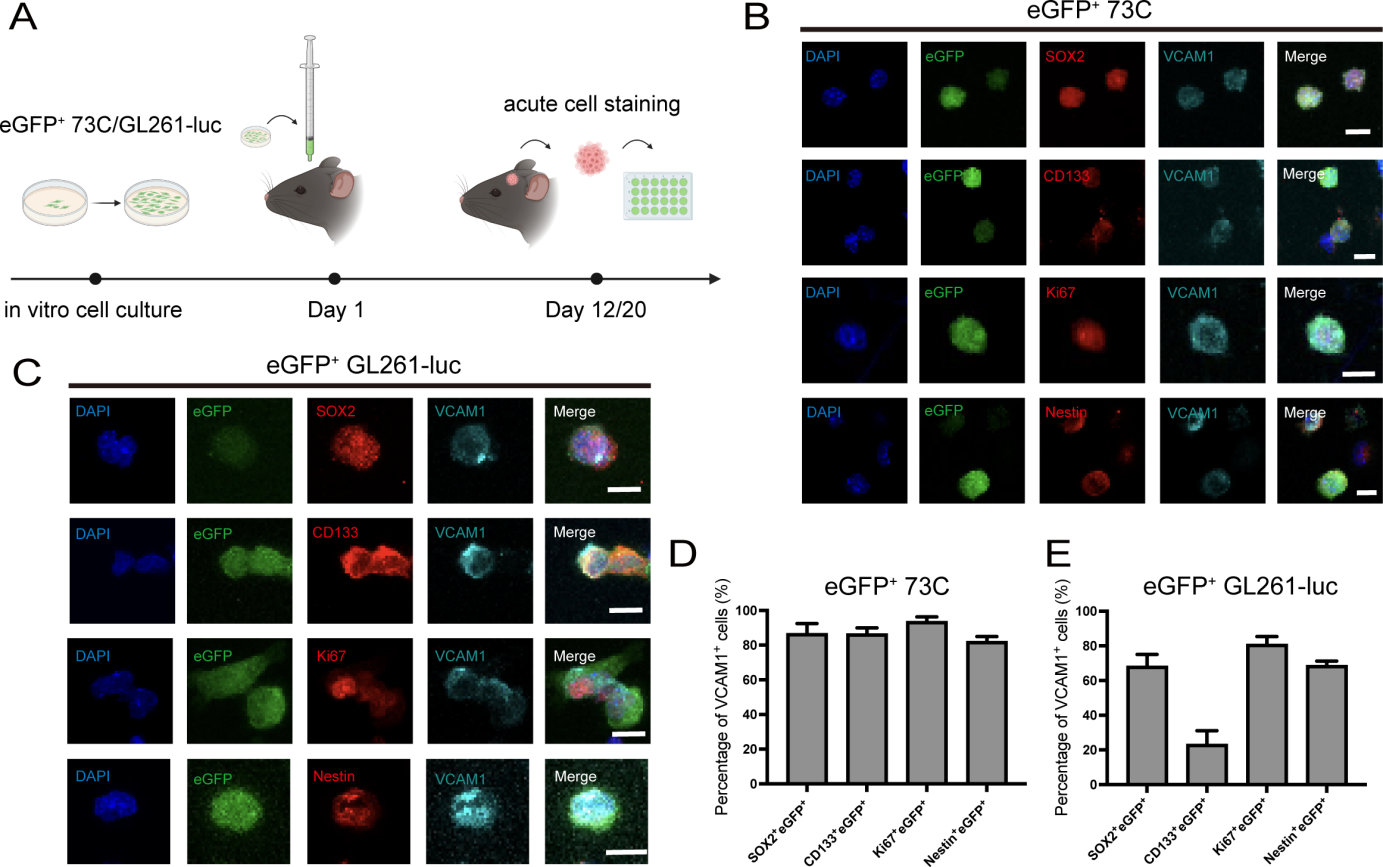
VCAM1 is expressed in glioma stem cells of eGFP+ 73C and eGFP+ GL261-luc tumor models in vivo. **(A)** Schematic timeline illustrating glioma cell implantation and acute cell staining. Gliomas were induced by intracranial implantation of 5,000–6,000 eGFP^+^ 73C or eGFP^+^ GL261-luc tumor cells in C57BL/6J mice. Acute cell staining was performed on day 12 post-implantation for eGFP^+^ 73C and on day 20 for eGFP^+^ GL261-luc. **(B)** Co-staining of VCAM1 with other glioma stem cell markers (SOX2, CD133, Nestin) and Ki-67 in acutely isolated eGFP**^+^** 73C glioma at 12 days post-implantation. Scale bar, 10 μm. **(C)** Co-staining of VCAM1 with other glioma stem cell markers (SOX2, CD133, Nestin) and Ki-67 in acutely isolated eGFP**^+^** GL261-luc glioma at 20 days post-implantation. Scale bar, 10 μm. **(D)** Quantitative analysis of the percentage of VCAM1-expressing cells in different glioma stem cell type populations in eGFP**^+^** 73C glioma. Data represent mean ± SEM (n = 6 biological replicates). Six randomly selected 500 × 500 μm regions were analyzed per sample. **(E)** Quantitative analysis of the percentage of VCAM1-expressing cells in different glioma stem cell type populations in eGFP^+^ GL261-luc glioma. Data represent mean ± SEM, n = 6 repeats. Six 500 μm×500 μm regions were randomly selected.

To further validate the enrichment of VCAM1 in GSLC populations *in vivo*, we acutely isolated cells from intracranial eGFP^+^ 73C and eGFP^+^ GL261-luc tumors (**Figure 1A**). Consistent with *in vitro* findings, VCAM1^+^ cells in both models highly co-localized SOX2, CD133, Nestin and Ki67 at 12 days (eGFP^+^ 73C) and 20 days (eGFP^+^GL261-luc) post-implantation (**Figures 1B, C**). Quantitative analysis revealed that in the eGFP^+^ 73C glioma model, VCAM1 was expressed in 86.500 ± 2.436% of SOX2^+^eGFP^+^ cells, 86.383 ± 1.465% of CD133^+^eGFP^+^ cells, 93.467 ± 1.164% of Ki67^+^eGFP^+^ cells and 81.983 ± 1.217% of Nestin^+^eGFP^+^ cells (**Figure 1D**). Similarly, in the eGFP^+^ GL261-luc glioma model, VCAM1 was expressed in 68.033 ± 2.885% of SOX2^+^eGFP^+^ cells, 23.117 ± 3.029% of CD133^+^eGFP^+^ cells, 80.700 ± 1.926% of Ki67^+^eGFP^+^ cells and 68.583 ± 1.097% of Nestin^+^eGFP^+^ cells (**Figure 1E**).

### VCAM1 expression in GSLCs of a primary glioblastoma model

**Figure 2:**
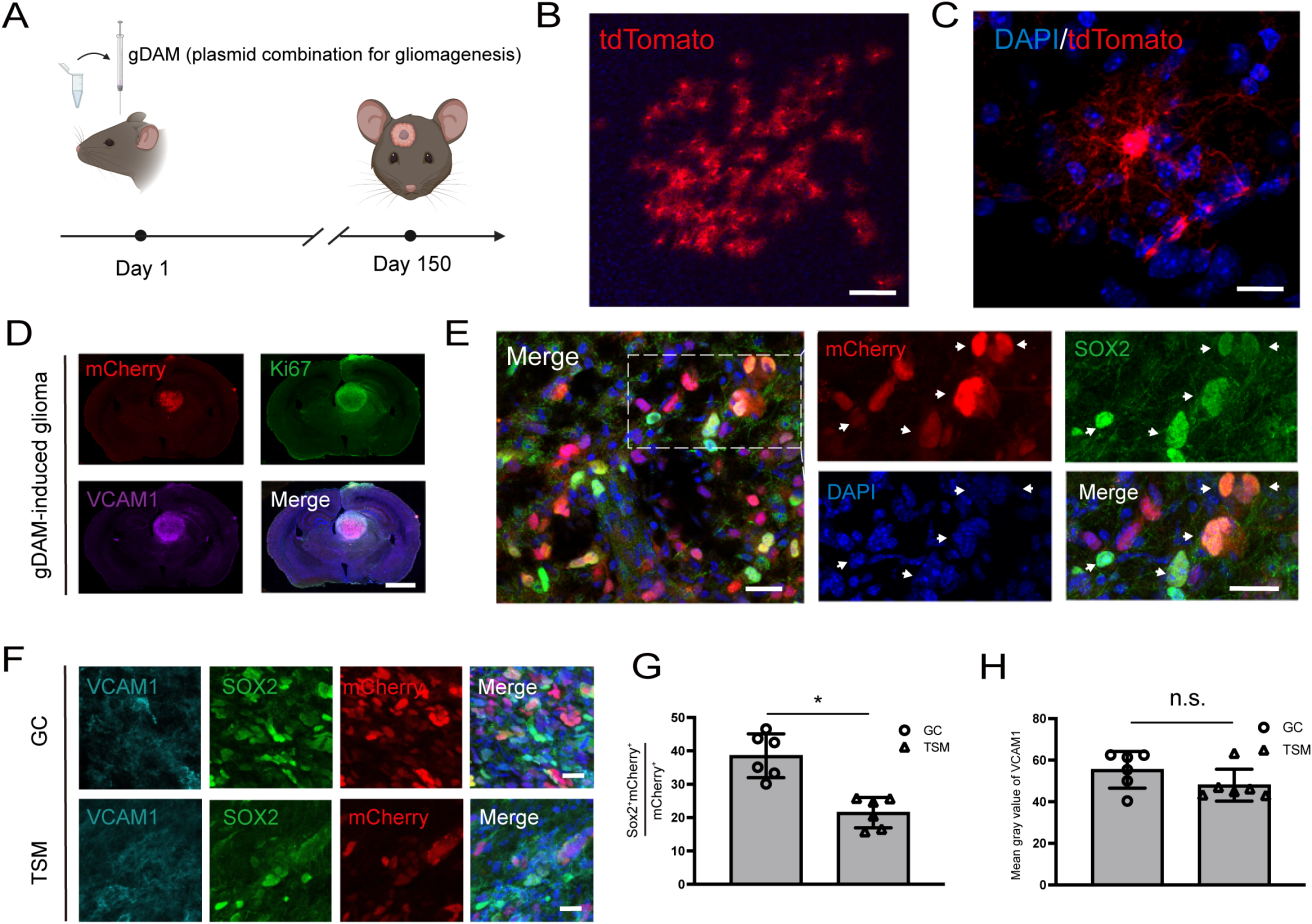
VCAM1 is expressed in glioma stem cell-like cells of gDAM-induced primary glioma *in vivo*. **(A)** Schematic timeline for the application of the gDAM method in C57BL/6J mice, involving the injection of a plasmid combination and proline mixture for gliomagenesis. **(B)** Labeling of tdTomato-expressing astrocytes in the brain of Ai14 mice using a Cre-expressing plasmid delivered via the gDAM method. Scale bar, 100 μm. **(C)** An astrocyte expressing tdTomato with characteristic endfoot structures. Scale bar, 20 μm. **(D)** gDAM-induced primary glioma observed at five months, showing immunostaining for Ki67 (green), VCAM1 (purple), and mCherry^+^ tumor cells (red). Scale bar, 2 mm. **(E)** SOX2-expressing mCherry^+^ tumor cells in a five-month-old primary glioma, indicated by yellow arrows. Scale bar, 20 μm. **(F)** Expression patterns of VCAM1, SOX2 and tumor cells in GC and TSM. GC, glioma core; TSM, tumor substantial margins. Scale bar, 20 μm. **(G)** The proportion of SOX2^+^mCherry^+^ cells relative to the total mCherry^+^ cells in the GC and the TSM. *p = 0.0313. Data are presented as mean ± SEM (n = 6 randomly selected fields derived from 3 mice per group). Statistical significance was determined by unpaired two-tailed Student’s t-test. **(H)** Relative fluorescence intensity of VCAM1 in the GC and TSM, based on randomly selected 200 μm × 200 μm regions. ^n.s.^*p* = 0.1563. Data are presented as mean ± SEM (n = 6 randomly selected fields derived from 3 mice per group). Statistical significance was determined by unpaired two-tailed Student’s t-test.

Having established that VCAM1 is highly enriched in the GSLC compartment of syngeneic transplantation models, we next sought to rule out the possibility that this expression pattern was merely an artifact of cell culture or the mechanical trauma of transplantation. Accordingly, we employed a primary glioma model generated using the gDAM method to induce astrocyte-derived tumors within the native, unperturbed microenvironment of immunocompetent mice (27). This model employs CRISPR/Cas9 to knock out *Trp53*, *Pten* and *Nf1*, combined with the transposon-mediated overexpression of EGFRvIII and an mCherry reporter (27). To validate the astrocyte-specific targeting, gDAM-mediated delivery of Cre recombinase into midbrain astrocytes of Ai14 reporter mice, induced tdTomato expression in cells with typical astrocytic morphology, including endfoot structures (**Figures 2B, C**).

Tumors developed in C57BL/6J mice by approximately five months (**Figures 2A, D**). Within these mCherry^+^ tumors, expression of Ki67, SOX2 and VCAM1 was substantially increased (**Figures 2D, E**). Further quantification indicated a significantly higher proportion of SOX2^+^ tumor cells (SOX2^+^mCherry^+^/total mCherry^+^) in the glioma core (GC, 38.54 ± 2.69%) compared to the tumor substantial margin (TSM, 21.50 ± 1.87%) (**Figures 2F, G**). Unlike the ubiquitous mCherry signal across the entire tumor mass, VCAM1 exhibited a heterogeneous and localized expression pattern (**Figure 2D**). This distribution reflects the fact that VCAM1 is specifically enriched in GSLCs, astrocytes, and vascular endothelial cells rather than the bulk tumor cells. Despite this macroscopic patchiness, quantification of the mean fluorescence intensity across randomly sampled microscopic fields showed that average VCAM1 levels were comparable between the GC and TSM sub-regions.(Relative intensity: 55.36 ± 3.60% in GC vs. 47.97 ± 3.14% in TSM) (**Figures 2F, H**).

### VCAM1 expression in astrocytes of adult mice

While our data confirmed VCAM1 expression within the tumor cell compartment, the progression of glioma relies heavily on interactions with stromal cells. To investigate whether stromal cells also contribute to the VCAM1 pool within the TME, we first needed to characterize the physiological distribution of VCAM1 in the adult mouse brain. To achieve this, we generated a VCAM1-CreER::Ai14 mouse strain (**Supplementary Figure 2A**). In this model, tdTomato^+^ cells comprised two primary populations: vascular-like and astrocyte-like cells. However, VCAM1 protein immunostaining co-localized exclusively with the vascular population, and no co-localization was observed in the astrocyte-like cells (**Supplementary Figures 3A, S3C, S3D**). Additionally, analysis of the Betsholtz Laboratory single-cell RNA sequencing database (http://betsholtzlab.org/VascularSingleCells/database.html) (28, 29) identified vascular fibroblast-like cells 2 (FB2), venous endothelial cells (vEC), astrocytes (AC), vascular fibroblast-like cells 1 (FB1), arterial endothelial cells (aEC) as the top Vcam1-expressing populations (**Supplementary Figure 2B**).

Furthermore, immunostaining for laminin confirmed the presence of vascular cells with the tdTomato^+^ population (**Supplementary Figure 3B**). Conversely, GFAP was not detected in the tdTomato^+^ cells (**Supplementary Figure 3C**). To definitively assess their astrocytic lineage, we generated ALDH1L1-eGFP::VCAM1-CreER::Ai14 mice. In this model, co-expression of tdTomato with the robust astrocyte marker ALDH1L1-eGFP confirmed that a small subset of tdTomato^+^ cells were indeed astrocytes. Thus, in the healthy adult cortex and hippocampus, VCAM1 is predominantly expressed by vascular endothelial cells, while astrocytic VCAM1 expression remains sparse and highly restricted (**Supplementary Figures 3A, S3B, S3E**).

### Astrocytic VCAM1 deletion prolongs survival in glioma mouse model

**Figure 3:**
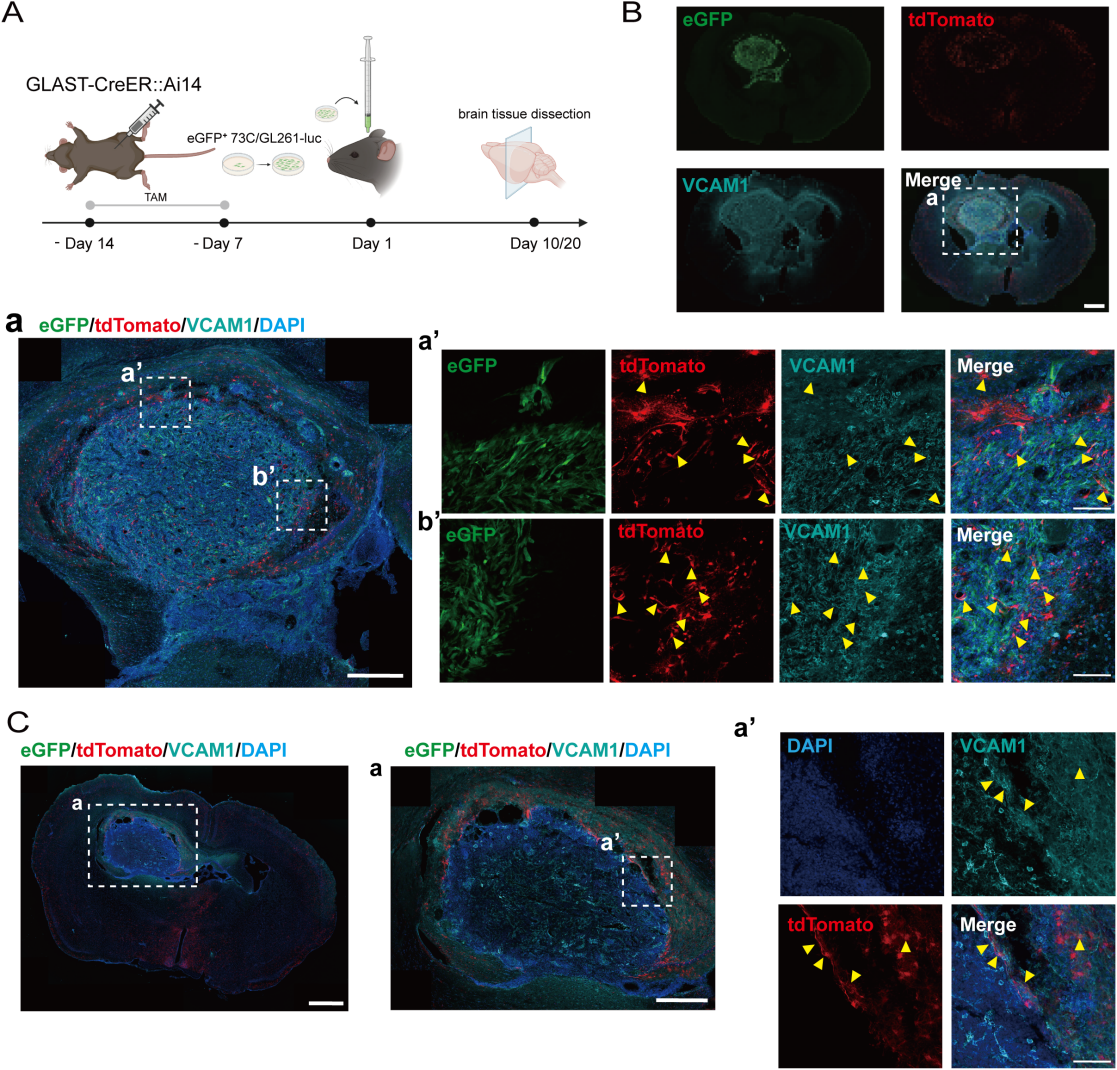
VCAM1 is expressed in a subset of GLAST+ astrocytes in glioma-bearing mice. **(A)** Experimental timeline for glioma cell implantation in GLAST-CreER::Ai14 mice. Mice received daily intraperitoneal injections of tamoxifen (TAM) for one week, followed by a one-week resting period. On day 1, 5,000-6,000 eGFP^+^ 73C or GL261-luc glioma cells were implanted beneath the hippocampus. Tumor-bearing mice were harvested on day 10 (eGFP^+^ 73C) or day 20 (GL261-luc). **(B)** Immunofluorescence staining of VCAM1 in eGFP^+^ 73C gliomas at day 10 post-implantation of GLAST-CreER::Ai14 mice. Scale bars: **(B)** 1 mm; (a), 500 μm; (a’, b’), 100μm. **(C)** Immunofluorescence staining of VCAM1 in GL261-luc gliomas at day 20 post-implantation of GLAST-CreER::Ai14 mice. Scale bars: **(C)** 1 mm; (a), 500 μm; (a’), 100 μm.

Although VCAM1 is predominantly restricted to vascular endothelial cells in the healthy brain, our immunofluorescence analysis (**Figures 3B, C**) revealed a distinct upregulation of VCAM1 in GLAST-positive astrocytes actively recruited to the tumor border. To determine whether this astrocytic VCAM1 functionally drives glioma progression, we utilized a genetic ablation approach. First, we generated GLAST-CreER::Ai14 mouse strain (**Supplementary Figure 4A**), which exhibited robust labeling of astrocytes in the cortex and sub-hippocampus, alongside some neuronal labeling in the hippocampus (**Supplementary Figure 4B**). We confirmed that VCAM1 is expressed in a subset of tumor-associated GLAST^+^ astrocytes at day 10 (eGFP^+^ 73C) and day 20 (GL261-luc) post-implantation (**Figures 3A–C**).

**Figure 4:**
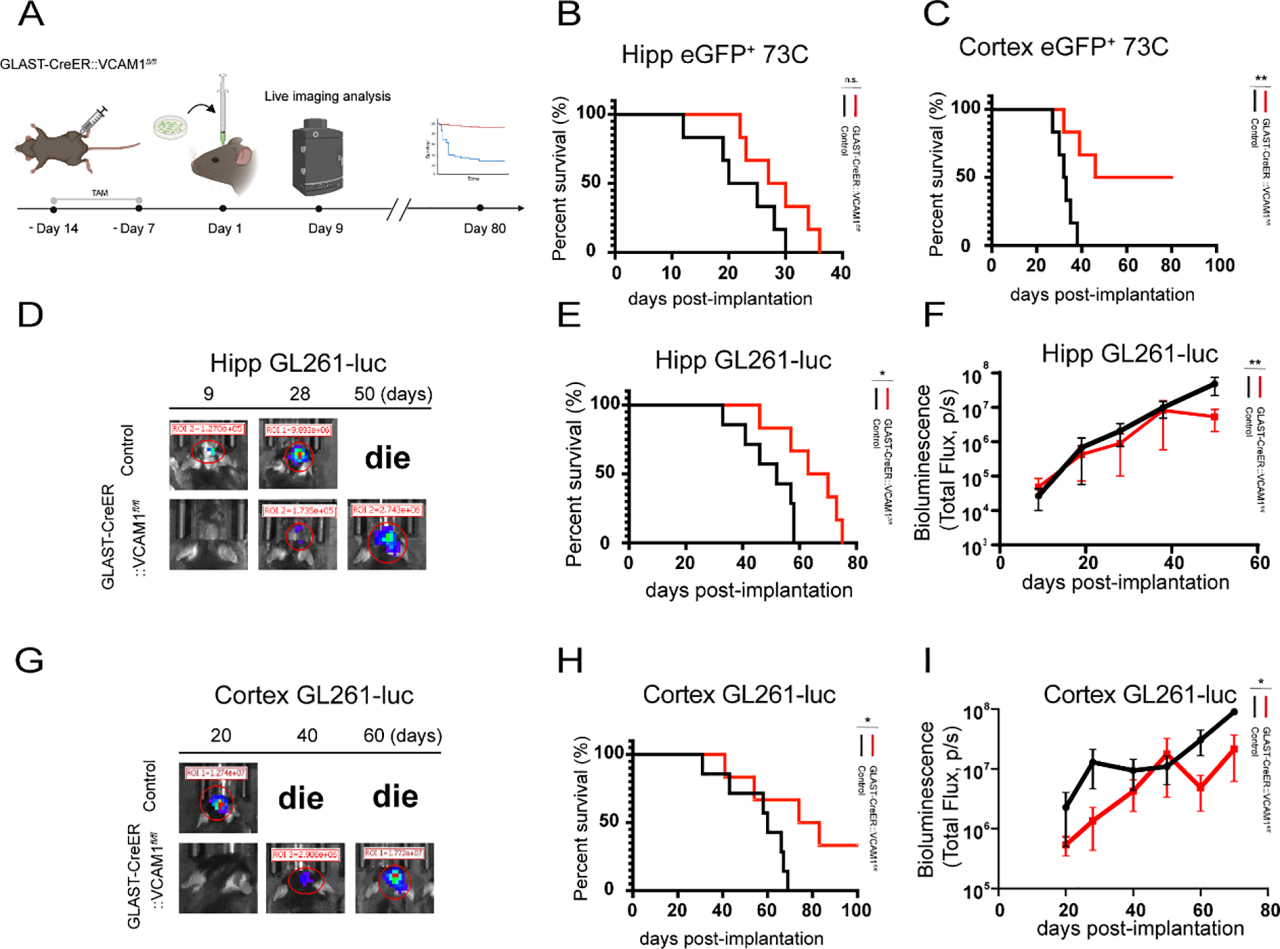
Knockout of VCAM1 in GLAST+ cells prolongs mouse survival. **(A)** Experimental timeline for glioma cell implantation in GLAST-CreER::VCAM1*^fl/fl^* and control mice. Mice received daily intraperitoneal injections of tamoxifen (TAM) for one week, followed by a one-week waiting period. On day 1, 5,000-6,000 tumor cells were injected into the cortex or below the hippocampus. Live imaging analysis was conducted at the predetermined points. **(B)** Kaplan-Meier survival curves of mice implanted with eGFP+ 73C tumor cells below the hippocampus. Control group (n = 6), GLAST-CreER::VCAM1fl/fl group (n = 6). p-value calculated using the log-rank Mantel-Cox test. Control vs GLAST-CreER::VCAM1fl/fl, “ns” indicates no statistical significance (*p* > 0.05). Here, *p* = 0.1209. **(C)** Kaplan-Meier survival curves of mice implanted with eGFP+ 73C tumor cells in the cortex. Control group (n = 6), GLAST-CreER::VCAM1fl/fl group (n = 6). p-value calculated using the log-rank Mantel-Cox test. Control vs GLAST-CreER::VCAM1fl/fl, **p = 0.0053. **(D)** Live imaging analysis of GL261-luc-bearing mice in hippocampus GLAST-CreER::VCAM1*^fl/fl^* and control groups at days 9, 28 and 50. **(E)** Kaplan-Meier survival curves of mice implanted with GL261-luc tumor cells below the hippocampus. Control group (n = 7), GLAST-CreER::VCAM1*^fl/fl^* group (n = 6). *p*-value calculated using the log-rank Mantel-Cox test. Control vs. GLAST-CreER::VCAM1*^fl/fl^*, *p = 0.0223. **(F)** Longitudinal quantification of tumor burden by bioluminescence imaging (Total Flux) in the Hippocampus GL261-luc models. Data are presented as mean ± SEM. Statistical significance was evaluated using Two-way repeated measures ANOVA. The asterisks indicate a significant interaction effect between time and genotype, **p = 0.0082. Data are presented as mean ± SEM (n = 6–7 mice per group). Statistical significance was evaluated using two-way repeated measures ANOVA. **(G)** Live imaging analysis of GL261-luc-bearing mice in cortex from the GLAST-CreER::VCAM1*^fl/fl^* and control groups at days 20, 40, 60. **(H)** Kaplan-Meier survival curves of mice implanted with GL261-luc tumor cells in the cortex. Control group (n = 7), GLAST-CreER::VCAM1*^fl/fl^* group (n = 6). *p*-value calculated using the log-rank Mantel-Cox test. Control vs. GLAST-CreER::VCAM1*^fl/fl^*, *p = 0.0465. **(I)** Longitudinal quantification of tumor burden by bioluminescence imaging (Total Flux) in the Cortex GL261-luc models. Data are presented as mean ± SEM. Statistical significance was evaluated using Two-way repeated measures ANOVA. The asterisks indicate a significant interaction effect between time and genotype, *p = 0.0106. Data are presented as mean ± SEM (n = 6–7 mice per group). Statistical significance was evaluated using two-way repeated measures ANOVA.

We next generated the GLAST-CreER::VCAM1*^fl/fl^* conditional knockout mice (**Supplementary Figure 5A**). We implanted 5,000-6,000 GL261-luc cells into the hippocampus or cortex of these mice and control littermates (GLAST-CreER::VCAM1*^fl/+^* and GLAST-CreER::VCAM1^+/+^), monitoring tumor growth longitudinally via *in vivo* bioluminescence imaging (IVIS) (**Figure 4A**). In the hippocampal model, astrocytic VCAM1 deletion significantly attenuated tumor growth (**Figure 4D**; **Supplementary Figure S5B**), and extended median survival from 52 days in control mice to 66.5 days (**Figure 4E**). A similar survival advantage was observed in the cortical model (median survival: 78.5 days vs. 60 days, **Figures 4G, H**). Notably, two astrocytic VCAM1 knockout mice with cortical tumors achieved long-term survival (> 100 days), and no macroscopic tumor burden (**Supplementary Figure 5C**). To dynamically quantify the tumor burden underlying this survival benefit, we longitudinally tracked the GL261-luc tumor expansion using IVIS. Consistent with the extended survival, astrocytic VCAM1 deletion mice exhibited a significantly suppressed tumor growth trajectory compared to control littermates. Two-way repeated measures ANOVA revealed a significant interaction between time and genotype in both the hippocampus model (**Figure 4F**) and the cortex model (**Figure 4I**), indicating that the rate of tumor expansion over time was substantially delayed following the loss of astrocytic VCAM1.

Furthermore, we extended our investigation to a more aggressive glioma model utilizing eGFP^+^73C cells. In the hippocampal implantation model, no significant difference in median survival was detected between astrocytic VCAM1 knockout mice (28.5 days) and controls (22.5 days) (**Figure 4B**). However, in the cortex implantation model, astrocytic VCAM1 deletion mice exhibited a significantly prolonged median survival of 63 days, compared to 32.5 days in controls (**Figure 4C**).

### The transcriptome reveals tumor microenvironment alterations following astrocytic VCAM1 deletion

**Figure 5:**
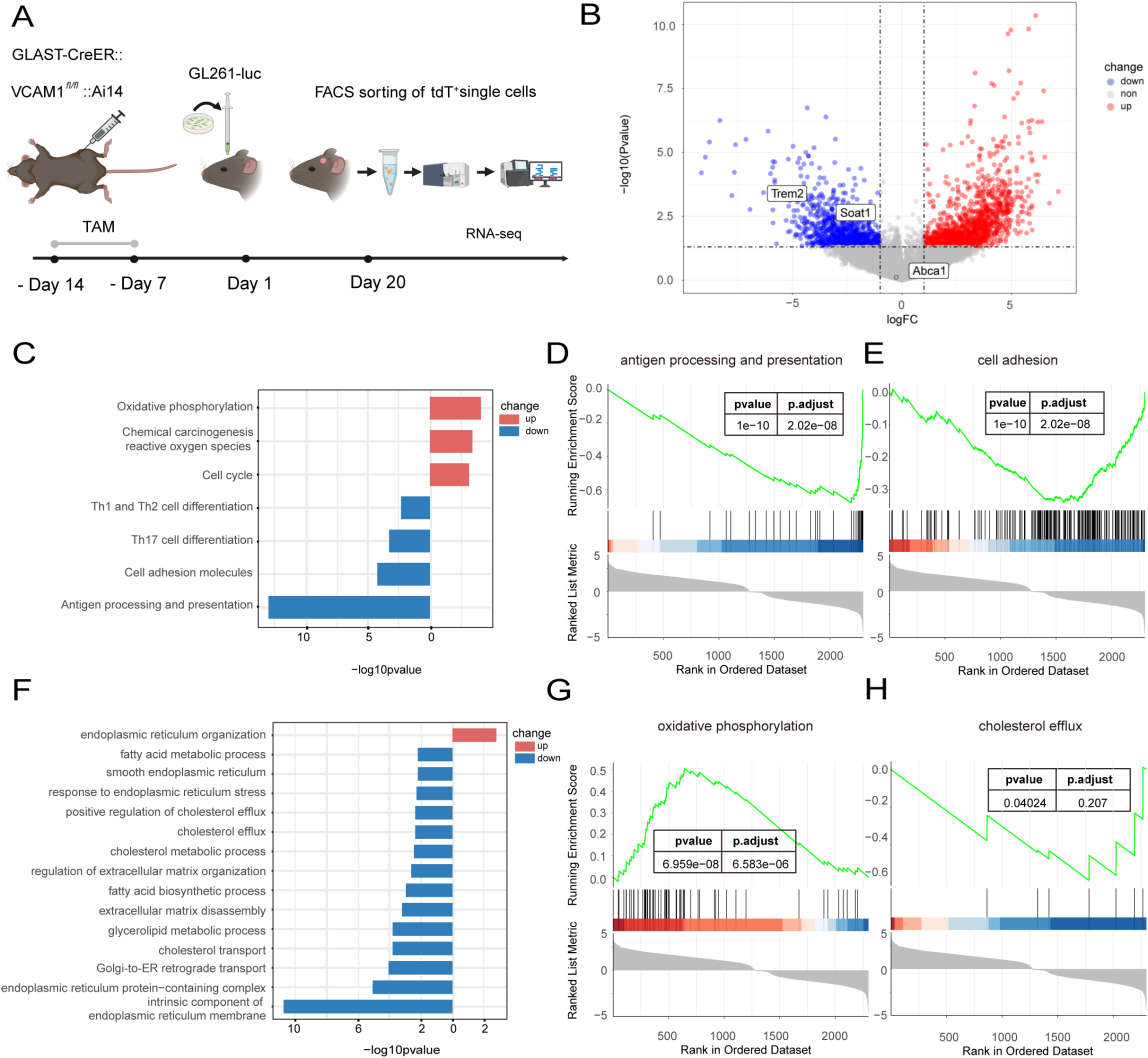
The RNA-seq reveals the changes of TME following astrocytic VCAM1 deletion. **(A)** The protocol of RNA-seq on tumor associated VCAM1-deficient GLAST positive astrocytes. n = 6 biological replicates per group (derived from 3 mice per group, 2 independent samples per mouse) were analyzed. **(B)** Differentially expressed genes in astrocytic VCAM1-deficient TME. **(C, F)** The KEGG and GO enrichment analysis of our inhouse dataset. **(D, E, G, H)** The GSEA analysis of differentially expressed genes identified several functional pathways, antigen processing and presentation, cell adhesion, oxidative phosphorylation, and cholesterol efflux.

To uncover the molecular mechanisms underlying this survival advantage, we performed transcriptomic profiling of the astrocytic niche. We implanted GL261-luc cells into GLAST-CreER:: VCAM1*^fl/fl^*::Ai14 mice (**Supplementary Figure 6A**) and isolated tumor-associated tdTomato^+^ astrocytes via FACS at day 20 post-implantation. Both the experimental (VCAM1-deficient) and control groups (VCAM1-proficient) consisted of three mice each, with two samples collected per mouse, resulting in 6 biological replicates per group (**Figure 5A**).

Principal component analysis (PCA) revealed clear separation between the two groups, indicating that VCAM1 deletion induces a consistent and substantial transcriptional shift in tumor-associated astrocytes (**Supplementary Figure 6B**). Specifically, tdTomato^+^ cells from the VCAM1-deficient group within the TME exhibited a distinct pattern, whereas control tdTomato^+^ cells displayed a more dispersed distribution.

Differential gene expression (DEG) analysis using the *limma* package identified 545 upregulated and 296 downregulated genes in VCAM1-deficient astrocytes (|log_2_ fold change| ≥ 1; *p* < 0.05) (**Figure 5B**). Functional enrichment analysis (|log_2_ fold change| > 0; *p* < 0.05) revealed significant downregulation in KEGG pathways related to cell adhesion molecules, T cell differentiation and antigen presentation (**Figures 5C–E**). Conversely, KEGG pathways related to oxidative phosphorylation, reactive oxygen species (ROS), and cell cycle showed significant upregulation in VCAM1-deficient astrocytes (**Figures 5C, G**). Furthermore, Gene Ontology (GO) analysis indicated a significant suppression of lipid and cholesterol metabolism, extracellular matrix synthesis and endoplasmic reticulum function in VCAM1-deficient astrocytes (**Figures 5F, H**).

However, expression of cholesterol transporter Abca1 remained unchanged, suggesting it is not a primary target affected by VCAM1 signaling in this context. Rather, the concurrent upregulation of OXPHOS and ROS pathways correlated with a targeted downregulation of specific lipid metabolism genes, such as Soat1 and Trem2 (**Figure 5B**), driving the overall shift in astrocytic cholesterol handling.

### VCAM1 expression correlates with survival and clinical characteristics in human gliomas

**Figure 6:**
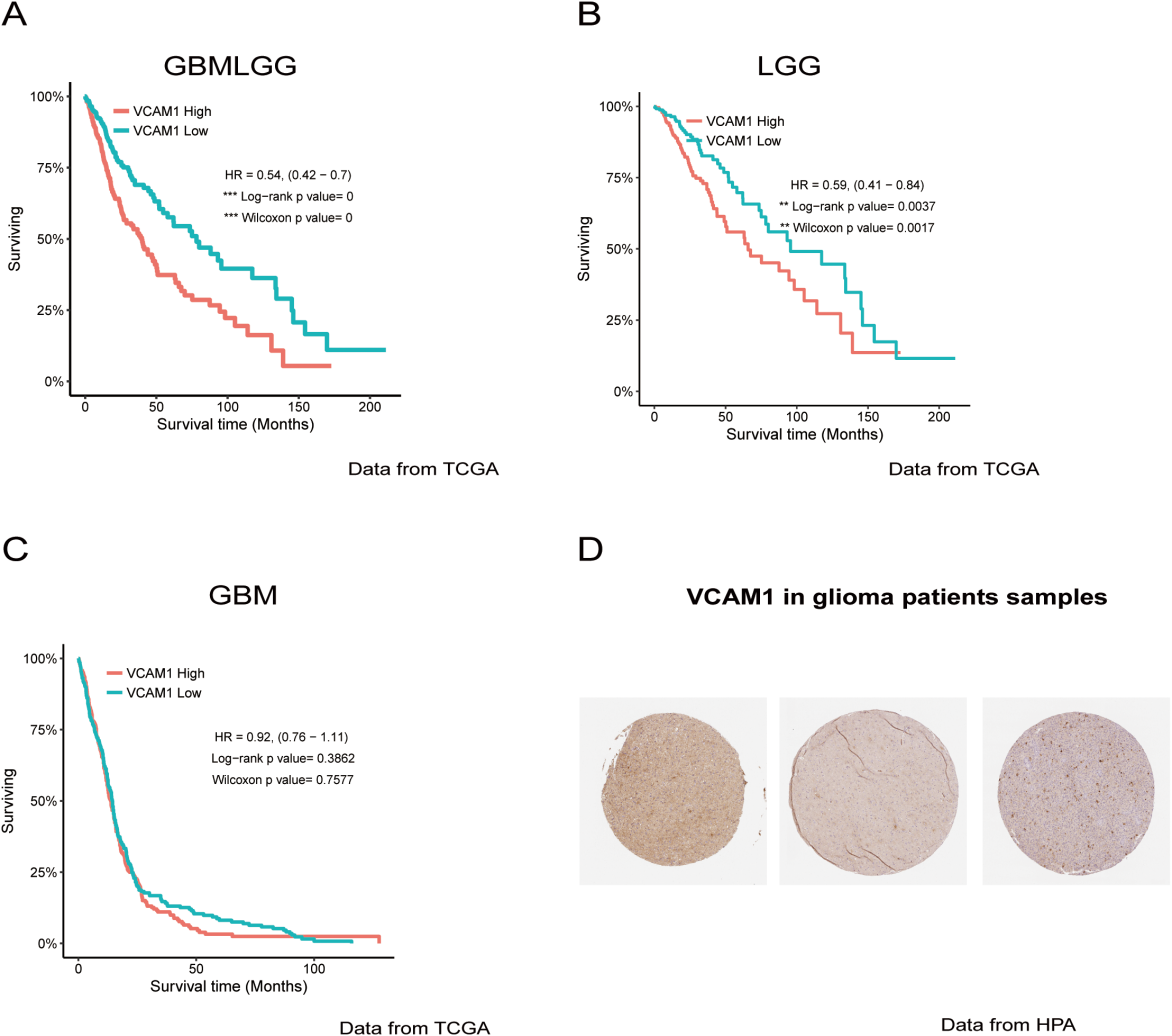
Clinical relevance of VCAM1 expression in human gliomas based on public database analysis. **(A)** Kaplan-Meier survival curve of patients with lower grade glioma and glioblastoma (GBMLGG) with different expression levels of VCAM1 in TCGA. The p-value was calculated using log-rank test (Mantel-Cox test). VCAM1>=9.605 (n=351) vs VCAM1<9.605 (n=343) *** *p* = 0.000001255. **(B)** Kaplan-Meier survival curve of patients with lower grade glioma (LGG) with different expression levels of VCAM1 in TCGA. The p-value was calculated using Mantel-Cox test. VCAM1>=9.243 (n=265) vs VCAM1<9.243 (n=263) ** *p* = 0.001199. **(C)** Kaplan-Meier survival curve of patients with glioblastoma (GBM) with different expression levels of VCAM1 in TCGA. The p-value was calculated using Mantel-Cox test. VCAM1>=10.25 (n=86) vs VCAM1<10.25 (n=80) ns *p* = 0.8022. **(D)** A 32-year-old female with moderate staining intensity of VCAM1 protein expression in LGG. Images were obtained from the Human Protein Atlas (HPA) database.

Our glioma models demonstrated that astrocytic VCAM1 is a critical vulnerability in the glioma microenvironment, dictating immune and metabolic crosstalk. To determine whether these preclinical findings translate to human pathology and hold prognostic value, we extended our analysis to clinical datasets. We performed survival analysis using data from The Cancer Genome Atlas (TCGA) database (https://portal.gdc.cancer.gov/; https://gliovis.bioinfo.cnio.es/) (30). This analysis revealed that lower VCAM1 expression was significantly associated with prolonged survival in patients with lower-grade glioma and glioblastoma (GBMLGG) (**Figure 6A**). Stratification by tumor grade demonstrated that this survival benefit was driven primarily by low-grade glioma (LGG) patients; VCAM1 expression did not significantly correlate with survival outcomes in the highly malignant glioblastoma (GBM) cohort (**Figures 6B, C**), directly mirroring the context-dependent resistance observed in our aggressive 73C mouse model. Using the UALCAN database (https://ualcan.path.uab.edu) demonstrated that VCAM1 is highly expressed in GBM (**Supplementary Figure 7A**). While VCAM1 expression did not significantly differ by sex, age group, or *TP53* mutation status (**Supplementary Figures 7B, S7D, S7E**), we observed significant variations across ethnic groups, with the highest expression in Caucasian cohorts (**Supplementary Figure 7C**).

Furthermore, evaluation of VCAM1 protein expression via The Human Protein Atlas (HPA) revealed variable staining intensities among glioma patients (**Figure 6D**). Consistent with our transcriptional data, a subset of patients exhibited moderate-to-low VCAM1 protein levels, underscoring the clinical heterogeneity of VCAM1 expression in human disease.

## Discussion

While the role of VCAM1 in facilitating leukocyte adhesion and promoting metastasis in peripheral cancers is well documented (13–15), its specific function within the highly specialized glioma microenvironment has remained elusive. Previous research in neuro-oncology has primarily viewed VCAM1 through the lens of tumor angiogenesis and endothelial cell biology (31–33). The primary novelty of our study lies in identifying astrocyte-derived VCAM1 as a critical, functionally distinct tethering node for glioma progression. To our knowledge, this is the first study to selectively ablate VCAM1 within the astrocytic niche (using an immunocompetent GLAST-CreER murine model) to evaluate its specific contribution to glioma growth *in vivo*. By shifting the focus from the vasculature to astrocytes, our work advances the current understanding of the TME. We provide direct *in vivo* evidence that expanding glioma cells rely heavily on astrocytic VCAM1 for structural anchoring.

Our initial characterization revealed that VCAM1 is expressed in GSLCs within GBM models. This observation, coupled with existing literature identifying VCAM1 as a marker for both embryonic and adult hippocampal neural stem cells (34, 35), suggests a potential functional relationship between GBM stem-like cells and neural stem cells. The consistent upregulation of VCAM1 across three distinct murine glioma models further underscores its potential significance in glioblastoma.

However, our results reveal a striking heterogeneity in the therapeutic potential of targeting astrocytic VCAM1.

This potential appears to be contingent upon both tumor location and the genetic background of the glioma. This discrepancy highlights the complexity of TME interactions in distinct brain regions. Several mechanisms may account for the resistance of hippocampal 73C tumors to VCAM1 depletion. First, anatomical constraints and distinct invasive properties of 73C cells may allow them to utilize VCAM1-independent mechanisms, such as disseminating along specific white matter tracts (24, 36). Second, regional heterogeneity dictates that hippocampal astrocytes might compensate for VCAM1 loss by upregulating alternative adhesion pathways. This dynamic likely differs from that in cortical astrocytes (37– 38). Third, the intrinsic genetic background significantly drives this heterogeneity. The 73C model is driven by specific aggressive mutations (BRAF alterations and Pten deletion). These genetic drivers fundamentally alter tumor-stroma interactions. This alteration potentially allows 73C cells to bypass specific VCAM1-dependent tethering mechanisms (39). Consequently, targeting VCAM1 cannot be applied as a universal monotherapy without considering regional microenvironments and molecular subtyping.

Importantly, the survival patterns observed in human glioma patients align with our *in vivo* functional data. In the TCGA dataset, lower VCAM1 expression correlates with longer overall survival in patients with low-grade glioma (LGG). However, this benefit is absent in patients with highly malignant glioblastoma (GBM). This clinical observation mirrors our murine models. Specifically, astrocytic VCAM1 deletion significantly extended survival in the less aggressive GL261 model. In contrast, it failed to alter survival in the highly aggressive, multi-mutant hippocampal 73C model. We propose that highly malignant tumors (such as human GBM and the murine 73C model) acquire VCAM1-independent invasive mechanisms or utilize alternative stromal tethers. Therefore, the efficacy of targeting VCAM1 depends heavily on intrinsic tumor grade and genetic background.

Furthermore, despite the abundant expression of vascular VCAM1, our results reveal that endothelial cells cannot functionally compensate for the loss of astrocytic VCAM1. We propose that this lack of compensation is driven by cell-type-specific metabolic coupling. Within the TME, astrocytes serve as a unique metabolic hub (11, 40, 41). As supported by our transcriptomic data (**Figure 5**), astrocytic VCAM1 acts as a critical bridge that anchors glioma cells. This close physical proximity facilitates essential metabolic cross-talk (24, 42), such as buffering oxidative stress (41) and supplying lipids and cholesterol (43, 44) vital for tumor growth. Although endothelial cells also express high levels of VCAM1, their metabolic and secretory profiles are distinctly different.

To investigate the underlying mechanisms, our transcriptome analysis suggests a dual impact on immune and metabolic pathways. We observed a significant downregulation of pathways related to cell adhesion, immune cell interactions and growth regulation. Specifically, the expression of key paracrine signaling genes decreased, such as Pdgfrb, Vegfa and Fgf1. We hypothesize that astrocytic VCAM1 knockout reduces immune cell infiltration. This reduction likely dampens pro-tumorigenic paracrine loops, effectively delaying tumor progression (45, 46).

Parallel to these immune and paracrine alterations, VCAM1 knockout in astrocytes led to the downregulation of cholesterol metabolism and transport pathways. Moreover, our transcriptome data revealed the upregulation of oxidative phosphorylation alongside an increase in ROS-related pathways. Elevated ROS contribute to damage to cellular organelles, such as the endoplasmic reticulum and Golgi apparatus (47). We hypothesize that this damage could subsequently inhibit astrocytic cholesterol metabolism.

However, we acknowledge several limitations in the current study. While our transcriptomic profiling strongly implicates alterations in astrocytic lipid metabolism and ROS pathways following VCAM1 deletion, direct functional validation of these downstream mechanisms is lacking. Specifically, determining exactly how VCAM1 anchors glioma cells to modulate astrocytic cholesterol efflux requires further targeted metabolic rescue experiments *in vivo*. Additionally, we propose hypotheses regarding the resistance of 73C hippocampal tumors to VCAM1 depletion. While concepts like regional astrocytic heterogeneity and specific genetic drivers are grounded in existing literature, they await rigorous experimental confirmation. Future studies should focus on dissecting these specific molecular pathways to fully uncouple the physical tethering function of VCAM1 from its metabolic signaling in the TME. Moving forward, cell-type-specific targeting of VCAM1 represents a promising therapeutic strategy, though its clinical translation will strictly require precise patient stratification based on anatomical location and malignancy grade.

## Conclusion

In conclusion, our study identifies astrocyte-derived VCAM1 as a critical tethering node that anchors glioma cells to the tumor microenvironment. By facilitating these physical interactions, VCAM1 supports tumor stemness and modulates local metabolic and immune responses, thereby promoting glioma progression. A noted limitation of this study is the reliance on transcriptomic data for downstream mechanistic insights; direct functional validation of this metabolic crosstalk remains a necessary next step. In the future, cell-type-specific targeting of VCAM1 signaling represents a promising therapeutic vulnerability. However, its successful clinical translation will strictly require precise patient stratification based on anatomical tumor location, molecular subtype, and malignancy grade.

## Data Availability

The datasets presented in this study can be found in online repositories. The names of the repository/repositories and accession number(s) can be found in the article/**Supplementary Material**.
