# Supplementary material for: Glia-derived VCAM1 promotes glioma progression
Source: Front Oncol. 2026 Apr 24;16:1802953. doi: 10.3389/fonc.2026.1802953 (PMC13152763; doi:10.3389/fonc.2026.1802953)
Supplement: Supplementary file 1 [file DataSheet1.pdf]

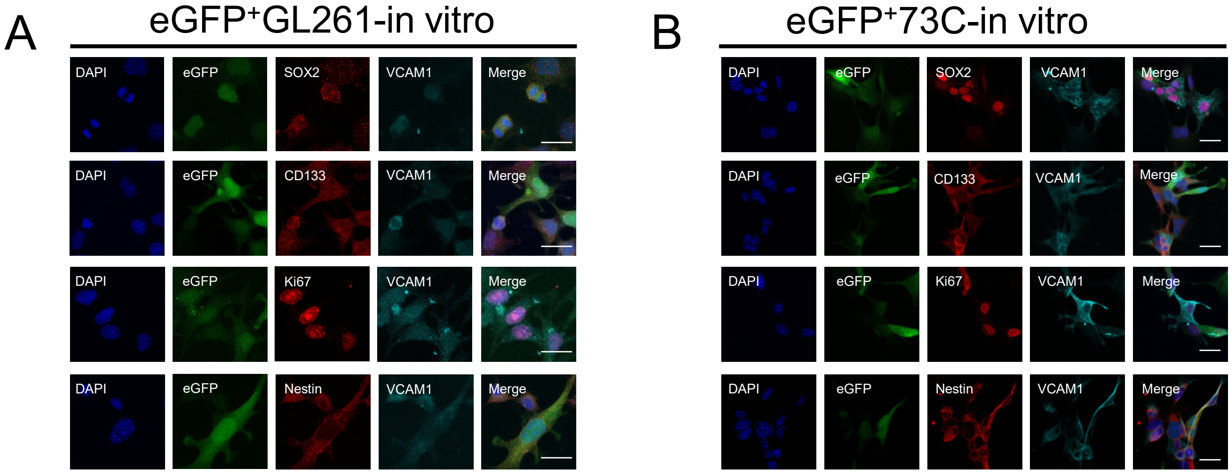

Supplementary Fig 1. VCAM1 is expressed in glioma stem cells of eGFP^{+} GL261-luc and eGFP^{+} 73C tumor cells in vitro.
(A) Co-staining of VCAM1 with other glioma stem cell markers (SOX2, CD133, Nestin) and Ki-67 in vitro culture cells of eGFP^{+} GL261-luc. Scale bar: 20 μm.
(B) Co-staining of VCAM1 with other glioma stem cell markers (SOX2, CD133, Nestin) and Ki-67 in vitro culture cells of eGFP^{+} 73C. Scale bar: 20 μm.

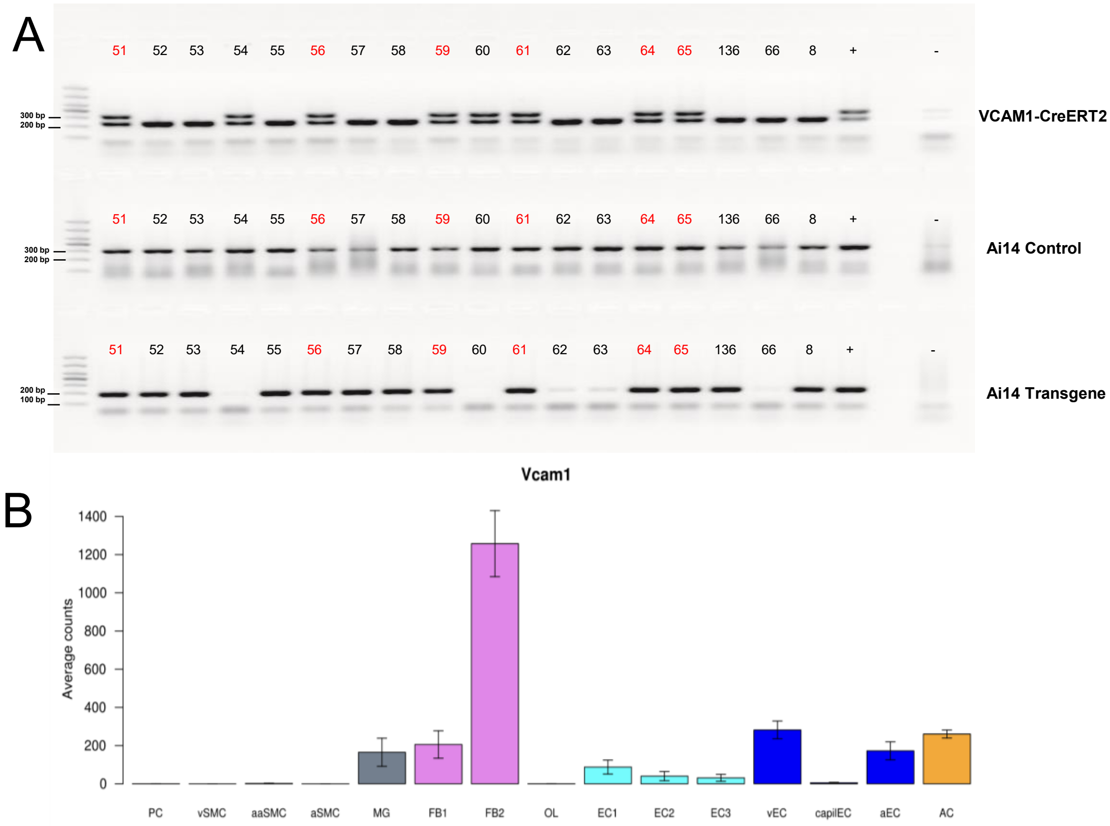

Supplementary Fig 2. Genotyping of VCAM1-CreER::Ai14 mice and VCAM1-expressing cells from Betsholtz Laboratory.

(A) Genotyping of VCAM1-CreER::Ai14 mice: The top row of bands represents the VCAM1-CreER mouse with the mut band and WT band (mut band:305bp, WT band: 222bp); the middle row represents the Ai14 mouse control band (297bp); and the bottom row represents the Ai14 mouse mut band (196bp). The mice with red numbers in the figure are the positive mice that successfully genotyped as VCAM1-CreER::Ai14.

(B) Average expression of VCAM1 in different cell types of vascular and vessel-associated cells. PC, pericytes; vSMC, vascular smooth muscle cells; aaSMC, arteriolar smooth muscle cells; aSMC, arterial smooth muscle cells; MG, microglia; FB,vascular fibroblast-like cells; 1,2, subtypes; OL, oligodendrocytes; EC, endothelial cells; vEC, venous endothelial cells; capilEC, capillary endothelial cells; aEC, arterial endothelial cells; AC, astrocytes.

Data from the interactive web portal(http://betsholtzlab.org/VascularSingleCells/database.html.) associated with Vanlandewijck, M., He,L et al. [Reference 28,29].

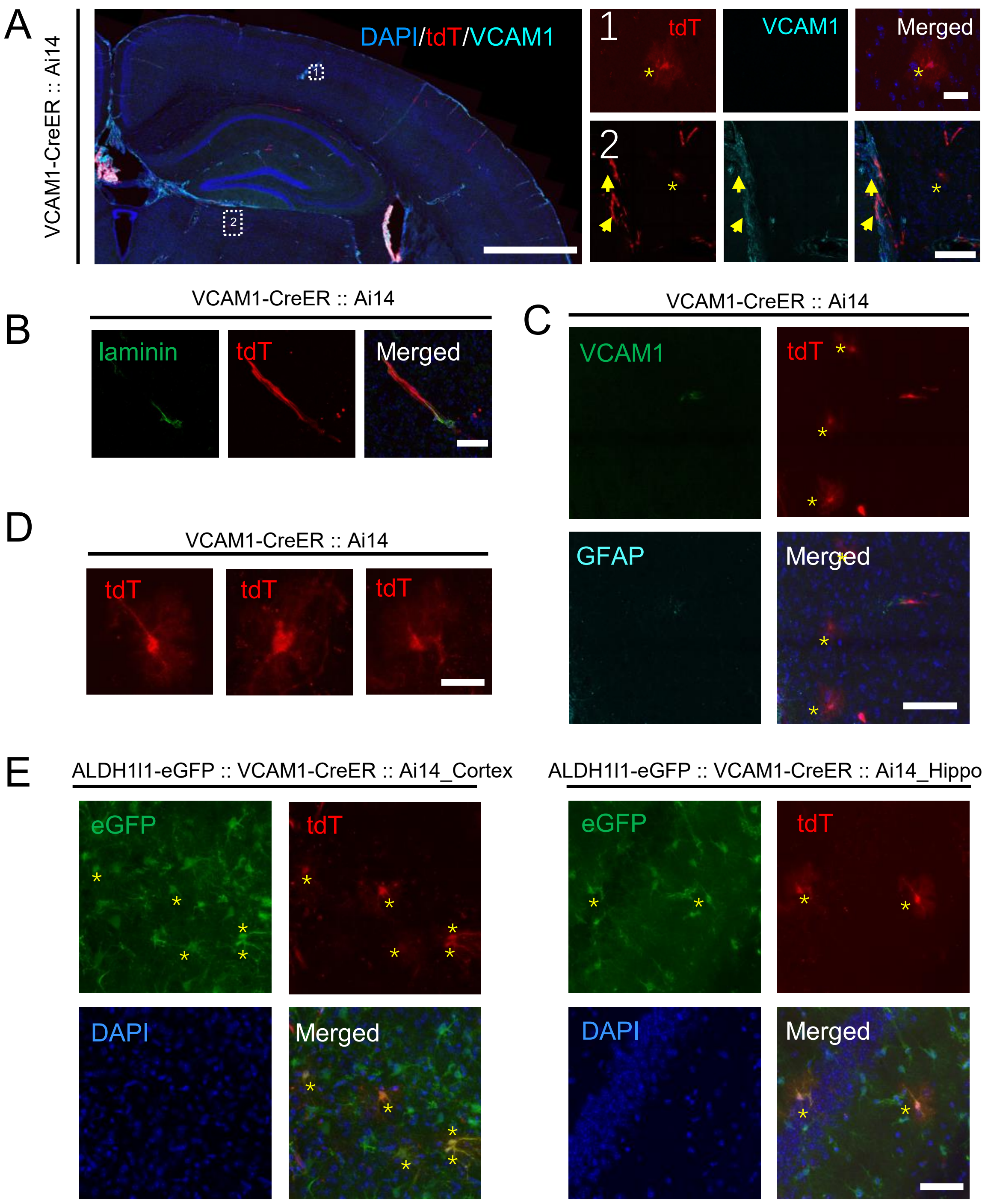

Supplementary Fig 3. VCAM1-expressing astrocytes in the cortex and hippocampus of adult mice.

(A) Quarter-brain slice of VCAM1-CreER::Ai14 mice with immunofluorescence staining for VCAM1 (cyan). Scale bar, 1 mm. (1) The white asterisk indicates an astrocyte. Scale bar, 20 μm; (2) The white arrow indicates vascular-type cells. Scale bar,100 μm.

(B) Immunofluorescence staining for laminin (green) in VCAM-CreER::Ai14 mice, showing tdTomato^{+} blood vessels. Scale bar, 100 μm.

(C) Immunofluorescence staining for VCAM1 (green) and GFAP (cyan) in VCAM-CreER::Ai14 mice, highlighting tdTomato^{+} astrocyte-like cells (white asterisk). Scale bar, 100 μm.

(D) tdTomato^{+} cells displaying typical astrocytic morphology. Scale bar, 10 μm.

(E) eGFP^{+} astrocytes and tdTomato^{+} cells (yellow asterisk) in ALDH1l1-eGFP::VCAM1-CreER::Ai14 mice. Scale bar, 50 μm.

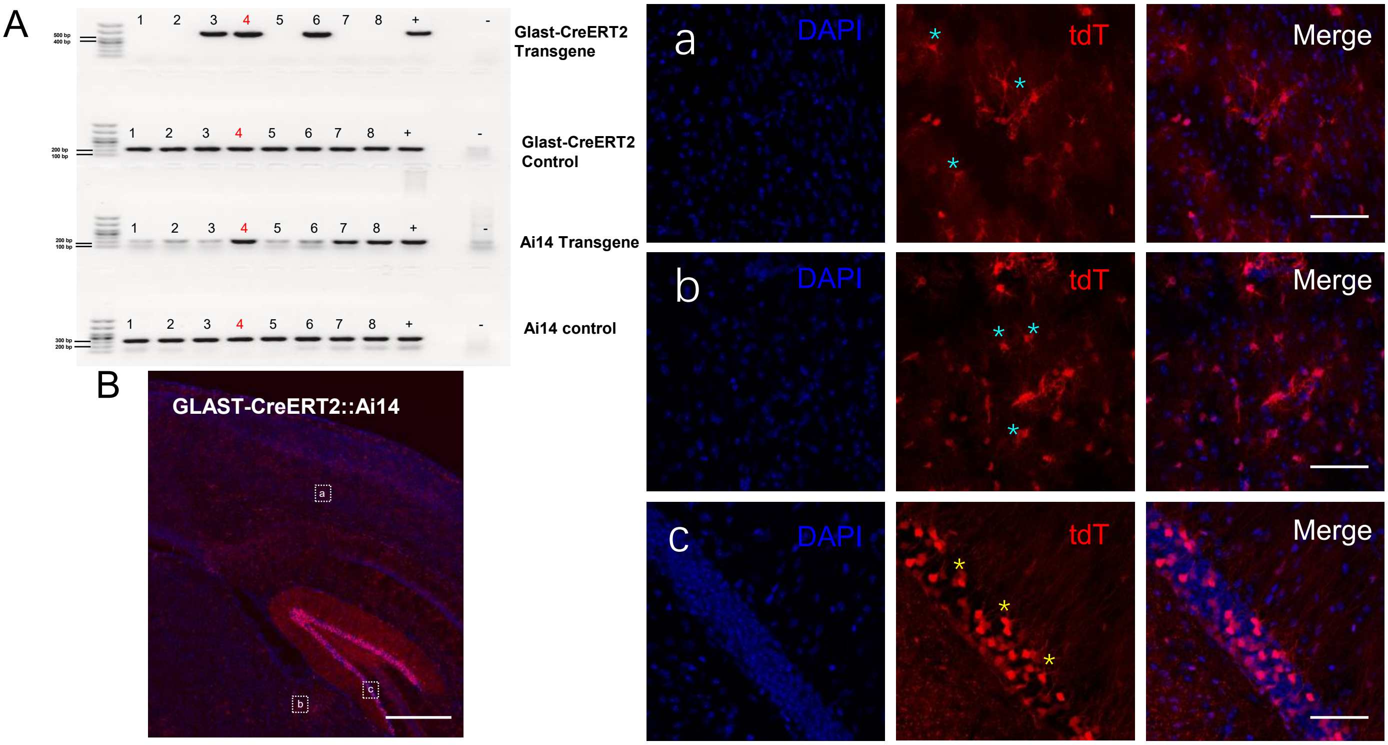

Supplementary Fig 4. Genotyping of GLAST-CreER::Ai14 mice and GLAST⁺ cells expression pattern

(A) Genotyping of GLAST-CreER::Ai14 mice: The top row of bands shows the GLAST-CreER mouse with the transgene band (600bp); the second row shows the GLAST-CreER wild-type mouse with the control band (200bp); the third row shows the Ai14 mouse mut band (196bp); the bottom row shows the Ai14 mouse control band (297bp). The No.4 mice in the figure successfully genotyped as GLAST-CreER::Ai14.

(B) $GLAST^{+}$ cells expression pattern of GLAST-CreER::Ai14 mice. Scale bar: 500 μm.

(a) cortex region (b) Sub-hippocampus region (c) Hippocampus region. Cells marked with blue asterisk are astrocytes, and yellow asterisk are neurons. Scale bar: 50 μm.

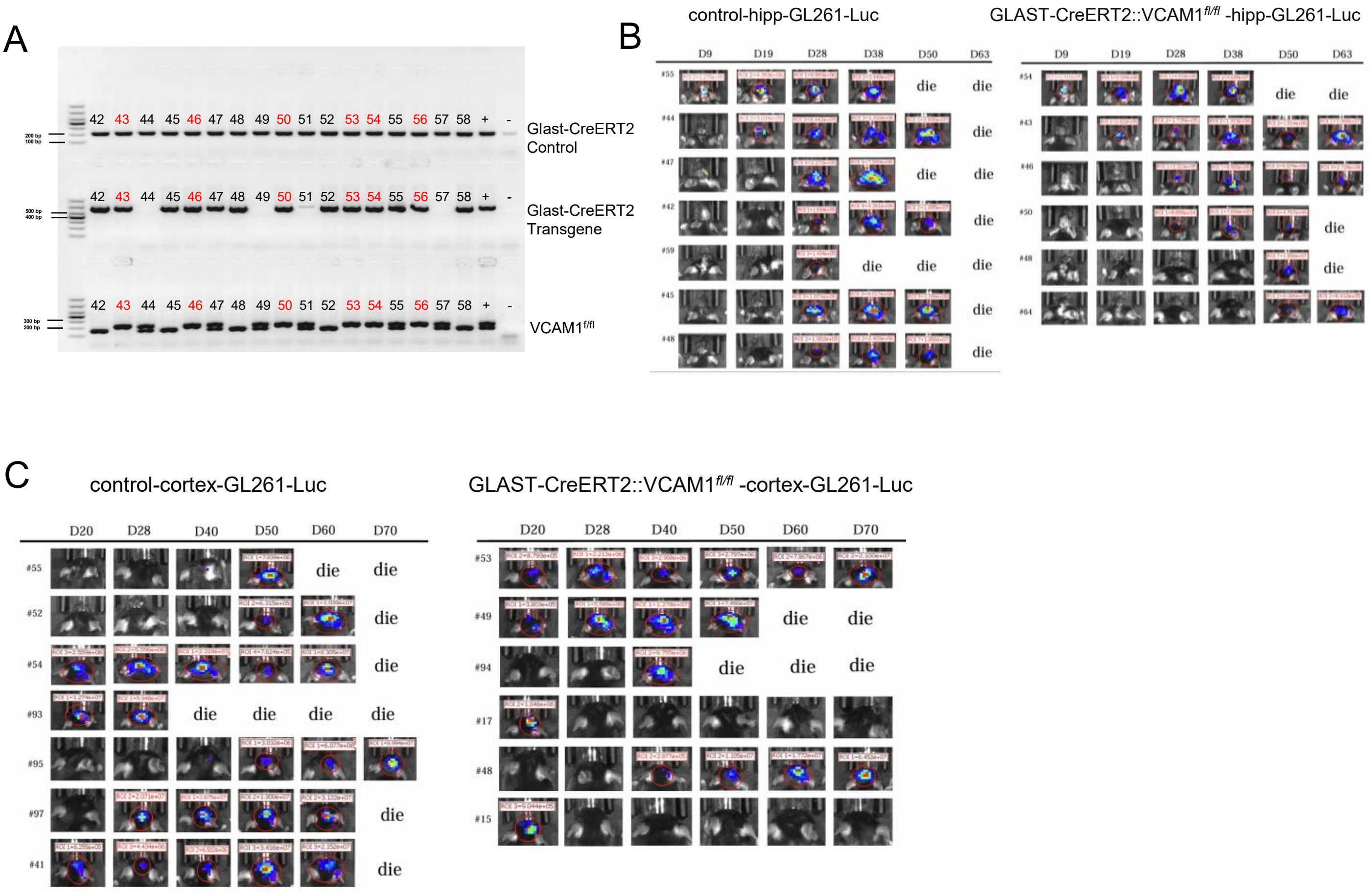

Supplementary Fig 5. Genotyping of GLAST-CreER::VCAM1$^{fl/fl}$ mice and glioma formation in GLAST-CreER::VCAM1$^{fl/fl}$ mice group and control group.

(A) Genotyping of GLAST-CreER::VCAM1$^{fl/fl}$ mice: The top row of bands shows the GLAST-CreER wild-type mouse with the control band (200bp); the middle row shows the GLAST-CreER mouse with the transgene band (600bp); and the bottom row shows the VCAM1$^{fl/fl}$ mice with the mut band and control band (mut band:226bp, control band: 162bp). The mice with red numbers in the figure are the positive mice that were successfully genotyped as GLAST-CreER::VCAM1$^{fl/fl}$ mice.

(B) Live imaging analysis of GL261-luc-bearing mice in hippocampus GLAST-CreER::VCAM1$^{fl/fl}$ group and control group at days 9, 19, 28, 38, 50, and 63.

(C) Live imaging analysis of GL261-luc-bearing mice in cortex GLAST-CreER::VCAM1$^{fl/fl}$ group and control group at days 20, 30, 40, 50, 60, 70.

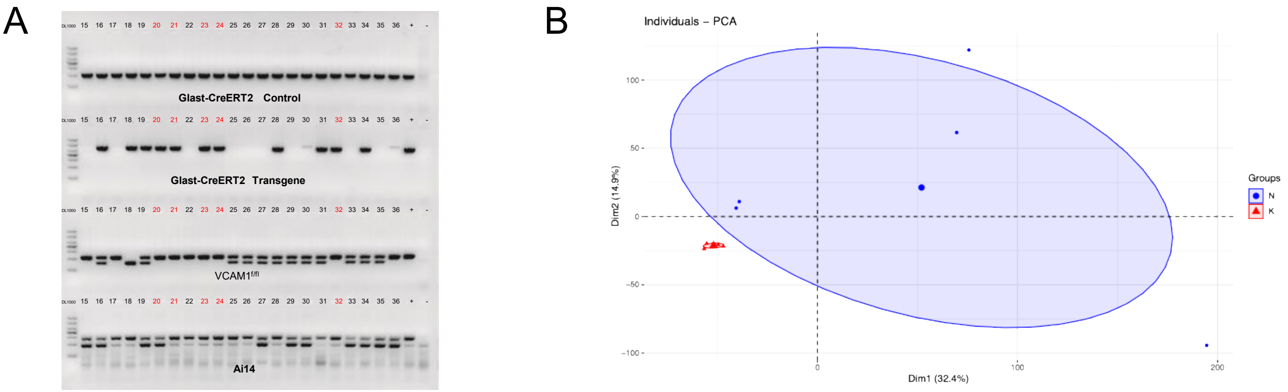

Supplementary Fig 6. Validation of Genotype for VCAM1_cKO mice and Principal Component Analysis (PCA) of RNA-seq datasets.

(A) In the genotyping of GLAST-CreER::VCAM1$^{fl/fl}$::Ai14 mice, the top row of bands displays a GLAST-CreER wild-type mouse with a control band (200 bp), the second row shows a GLAST-CreER mouse with a transgene band (600 bp), the third row presents VCAM1$^{fl/fl}$ mice with both a mutant band (226 bp) and a control band (162 bp), and the bottom row illustrates an Ai14 mouse with a control band (297 bp) and a mutant band (196 bp); the mice labeled with red numbers in the figure are those that were successfully genotyped GLAST-CreER::VCAM1$^{fl/fl}$::Ai14 mice.

(B) Principal component analysis (PCA) of transcriptomic profiles depicting distinct clustering of Normal (N) and Vcam1$^{-/-}$ (K) groups across the first two principal components (PC1: 32.4% ; PC2: 14.9%).

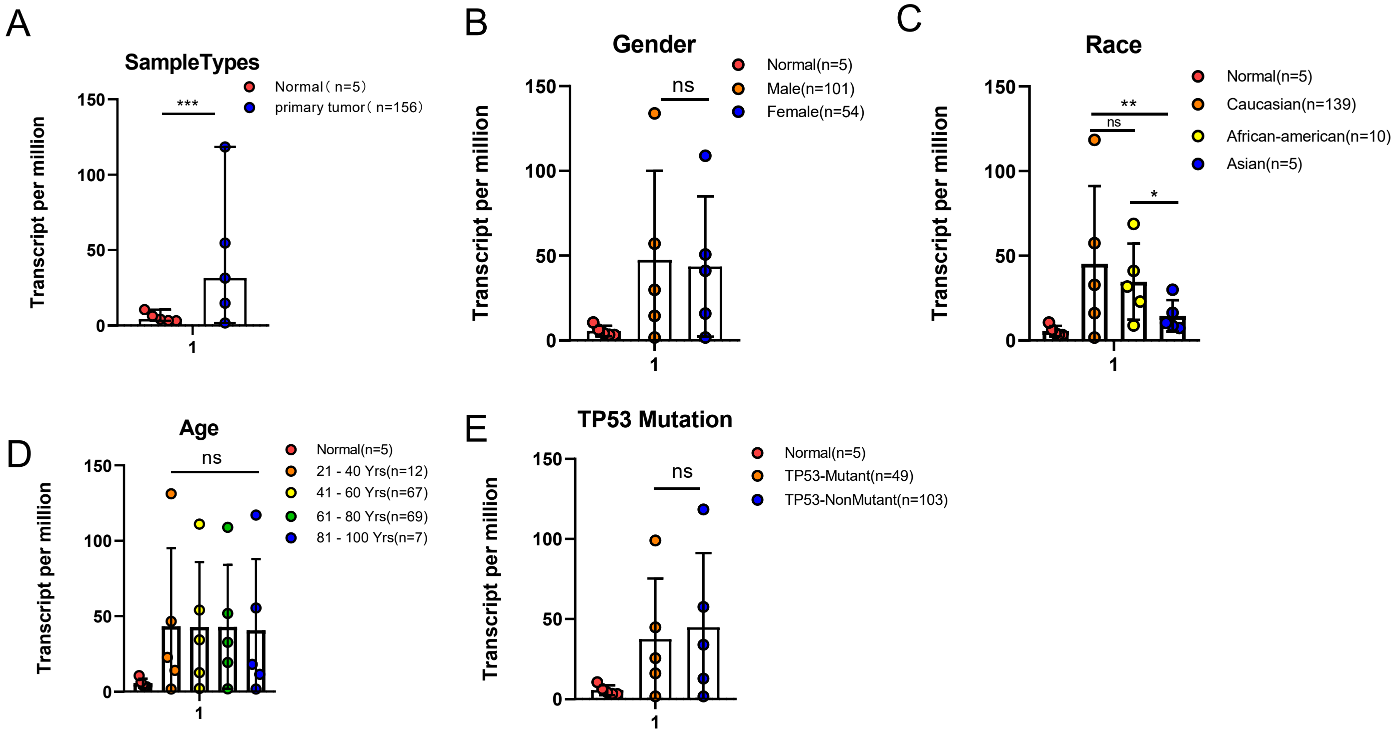

Supplementary Fig 7. Differential expression of VCAM1 in glioma patients based on clinical and demographic factors (Data from UALCAN).

(A) Expression levels of VCAM1 in patients with primary glioma (n=156) compared with normal samples (n=5) from UALCAN. The *p*-value was calculated using an unpaired t-test. $^{***}p$=0.0001.

(B) Expression levels of VCAM1 in patients with glioma of male (n=101) and female (n=54). The *p*-value was calculated using a paired t-test. ^{ns}p>0.9.

(C) Expression levels of VCAM1 in patients with glioma of Caucasian (n=139), African-American (n=10) and Asian (n=5). Caucasian vs. Asian, $^{**}p$=0.0083, African-American vs. Asian, $^{*}p$=0.0302, Caucasian vs. African-American, ^{ns}p=0.6250.

(D) Expression levels of VCAM1 in patients with glioma in different age group. 21-40 years (n=12); 41-60 years (n=67); 61-80 years (n=69); 81-100 years (n=7), ^{ns}p=0.3756

(E) Expression levels of VCAM1 in patients with glioma with wild-type (n=103) and mutant TP53 (n=49), ^{ns}p>0.9.
